# Advancements in macroinvertebrate-based river bioassessment research in the Afrotropical region: review and steps towards a regional framework

**DOI:** 10.1007/s10661-025-14272-3

**Published:** 2025-07-21

**Authors:** Priscilla Wagari Mureithi, Amon Aine, Rose Basooma, Judith Namumbya, Florence Nansumbi, Mourine Jessie Yegon, Harald Meimberg, Wolfram Graf

**Affiliations:** 1 Institute of Hydrobiology and Aquatic Ecosystem Management, Department of Ecosystem Management, Climate and Biodiversity, University of Natural Resources and Life Sciences Vienna (BOKU), Gregor-Mendel-Straße 33, 1180 Vienna, Austria; 2 Institute for Integrative Nature Conservation Research, Department of Ecosystem Management, Climate and Biodiversity, University of Natural Resources and Life Sciences Vienna (BOKU), Gregor-Mendel-Straße 33, 1180 Vienna, Austria; 3 Department of Biological Sciences, Faculty of Science, Egerton University, Egerton-Njoro, 536-20115 Kenya; 4 Department of Natural Resources Economics, Faculty of Natural Resources and Environmental Sciences, Namasagali Campus, Busitema University, P. O. Box 17, Tororo, Uganda; 5 Department of Biological Sciences, Faculty of Science, Kyambogo University, P. O. Box 1, Kampala, Uganda; 6 Wassercluster Lunz - Biological Station, Dr. Carl Kupelwieser-Prom. 5, Lunz Am See, 3293 Austria

**Keywords:** Rivers, Bioassessment, Macroinvertebrates, East Africa, Tropical, Ecological integrity

## Abstract

Rivers and streams are critical components of ecosystems, providing essential resources and supporting diverse aquatic life. Rivers have different physical and chemical characteristics based on geology, latitude, longitude, and climate. These factors influence the aquatic taxa composition and their sensitivities to ecosystem degradation gradients. Assessing the health of these aquatic ecosystems is vital for effective conservation and management. In recent years, there has been growing interest among East African countries to develop and adapt nationwide frameworks for the assessment of ecological integrity based on benthic macroinvertebrates as bioindicators of streams and rivers. Nevertheless, current initiatives have not resulted in binding policy and standardized protocol. Scholars realize the lack of baseline research in the spatial and temporal variation of abiotic and biotic components of East African Rivers, which are the foundation for development and integration of biomonitoring techniques. This review article explores the commonly used bioassessment techniques applied to river ecological integrity assessment, their strength, challenges, and opportunities in their application in East African contexts. It builds on these findings to propose a systematic pathway that begins at the national level but is standardized towards the development of a comprehensive policy-level bioassessment framework for the Afrotropical region.

**Supplementary Information:**

The online version contains supplementary material available at 10.1007/s10661-025-14272-3.

## Introduction

Freshwater ecosystems are habitats to approximately 10% of the earth’s biodiversity (Strayer & Dudgeon, 2010) and are among the most vulnerable ecosystems due to climate change and anthropogenic pollution (Hellawell, 2012). These stressors are anticipated to proliferate further due to the rising population, which inflicts an ever-stronger footprint on natural ecosystems (Tittensor et al., 2014). However, despite ecological stressors, there is a paucity of nationally or regionally standardized and reliable bioassessment tools, which hinders effective and integrated policymaking and management. In the recent past, there has been a rising interest among decision makers to use bioindicator-based frameworks in the assessment and prediction of the rising interlinked ecological stressors (Hoffmann et al., 2014).

Emerging attention on river health assessment techniques to support physico-chemical methods has led to development of diverse rapid assessment methods using aquatic organisms (Moog et al., 2018). The most used organisms include benthic macroinvertebrates, fish, macrophytes and periphyton (Aazami et al., 2015; Masouras et al., 2021). However, the choice of an indicator depends on ecological characteristics, sensitivity to ecological stressors, abundance, distribution, mobility, and the ability for standardization and quantification (Manickavasagam et al., 2019). Benthic macroinvertebrates are ubiquitous, sedentary, and sensitive to environmental changes and therefore, are the most dominantly used in river bioassessment (Sumudumali & Jayawardana, 2021).

Currently in the Afro-tropics, there are limited systematic approaches documented and nationally adopted for riverine bioassessment despite the region being endowed with vast freshwater resources. Furthermore, the few developed indices have not been integrated into national and regional policy. This limits efforts to properly manage and conserve these aquatic ecosystems from the pressures that degrade them.

Past bioassessment studies have recommended the development of region-specific bioassessment systems and indices in the Afro-tropics (Elias et al., 2014a, b; Kaaya et al., 2015; Ochieng et al., 2019; Maese & Dalu, 2024). This is because the existing indices being used are borrowed or widely adopted from non-tropical regions. The regional differences in geology, latitude, altitude, and climate shape unique physical, chemical and biological characteristics of river systems (Elias et al., 2014a, b). For macroinvertebrate-based river bioassessment, the regional differences affect the performance, functionality, compatibility, and reliability of the indices. Therefore, there is a risk of unreliability when adopting, improving, and applying non-tropical bio-assessment methods to evaluate ecological integrity of Afro-tropical rivers and streams.

There is a general gap in the development of systematic standardized protocols for East African regional bioassessment frameworks. This review article aims at (i) reviewing the commonly used bioassessment indices and protocols applied to assess river ecological integrity, their strengths, challenges, and opportunities in their application in East African context and (ii) providing a standardized systematic procedure for the development of macroinvertebrate-based river assessment systems and their integration into East African regional river assessment and legislation frameworks for sustainable use of freshwater systems.

## Methodology

**Fig. 1:**
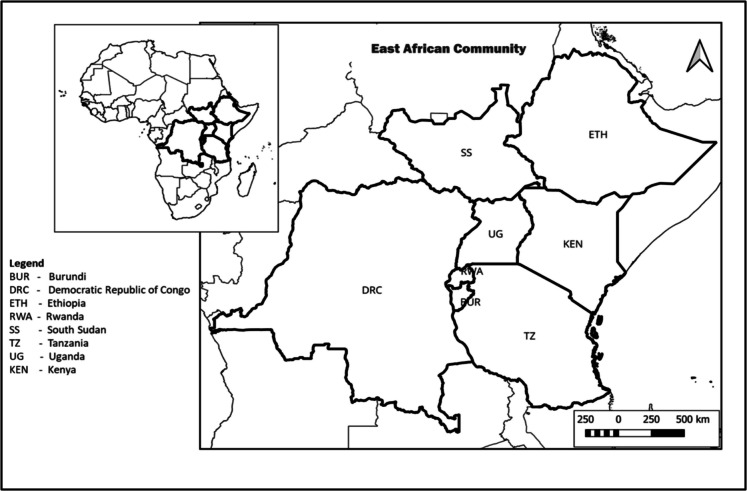
Case study countries members of the East African Community member countries within the Afrotropical region

**Fig. 2:**
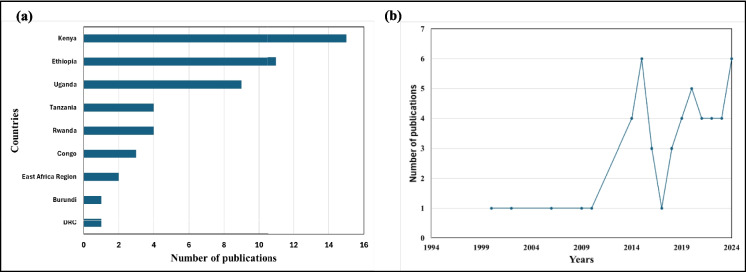
**a** Distribution of published articles on river or stream bioassessment using macroinvertebrates within the East African region. **b** Frequency and trend of scientific articles on river bioassessment and related topics from 2000 to the present. In the graph, “East African region” refers to studies covering the broader East Africa zone without focusing on specific countries

We conducted a literature review to identify commonly used macroinvertebrate bioassessment tools and related aspects of river monitoring within East Africa since 2000 (Fig. 1). This comprehensive review involved searches across multiple databases, including Google Scholar, ScienceDirect, Web of Science, and ResearchGate. The search was narrowed down using the following keywords: rivers, bioassessment, bioindicators, macroinvertebrates, Afrotropical, ecoregions, and river ecosystems. With Scopus advanced search on titles, keyword and abstract using the algorithm (Bioassessment OR Biomonitoring) AND (Macroinvertebrates OR Benthic* OR Functional*) AND (River* OR Stream*) AND (“East Africa” OR Africa OR Tanzania OR Kenya OR Uganda OR Rwanda OR Burundi OR “Democratic Republic Of Congo” OR “South Sudan” OR Ethiopia AND NOT “South Africa”)) And Exclude (Exactkeyword, “Africa, Western”) Or Exclude (Exactkeyword, “Asia, Southeastern”) Or Exclude (Exactkeyword, “Australasia”) Or Exclude (Exactkeyword, “Australia”) Or Exclude (Exactkeyword, “Asia”) Or Exclude (Exactkeyword, “United States”) Or Exclude (Exactkeyword, “West Africa”) (Fig. 2). The review further employed in-depth review and analysis of information from different peer-research reports, conference proceedings and reviewed publications that met the scope of the study.

### Common indicator groups used in assessment of river ecological integrity

Assessing the integrity of aquatic ecosystems is an ongoing field of research, with ecological indicators being key diagnostic tools. These indicators are used to evaluate river health by comparing current conditions to reference sites (Karr, 1999; Verdonschot et al., 2020). Indicators enable understanding the responses of different components of the ecosystem to specific stressors (Young et al., 2006). Indicators are categorized as either structural or functional indicators to recognize that the ecosystem is characterized by both its structure and function. Structural indicators are those which measure the setting and composition of the ecosystem—that is, its physical, chemical, or biological components (Matthews et al., 1982; Sandin & Solimini, 2009). Monitoring of ecosystem structure is commonly represented by the measurement of specific organisms’ biophysical characteristics (Matthews et al., 1982), wider species richness and density, and nutrient concentrations (Young & Collier, 2009; Estevez et al., 2017).

Functional indicators quantify the rate or relative importance of aquatic processes (Von Schiller et al., 2017; Young et al., 2006). They are categorized into four groups, i.e., measures of process rates such as rates of litter decomposition, states linked to processes such as the amount of biomass, status and composition of functional traits underlying processes such as macroinvertebrate functional feeding groups and food web level process indicators (Harrison et al., 2021). Stream metabolism and organic matter decomposition are the most used functional indicators in assessing ecosystem health (Ferreira et al., 2020). These processes have shown sensitivity to anthropogenic stressors, particularly land use and associated impacts, and can be predicted between pristine and disturbed systems (Sitati et al., 2021).

Direct measures of functional indicators are integrative and can be used to measure characteristics of multiple habitats within streams simultaneously and are more geographically independent because they do not rely on a specific group of species (Feio et al., 2010). They are also beneficial where taxonomic expertise is lacking (Young et al., 2008) and therefore can be used to substitute taxonomy-based assessment systems. However, there is a lack of standardized protocols to incorporate direct measures of functional indicators into regular monitoring (von Schiller et al., 2017). Additionally, their widespread adoption is hampered by insufficient knowledge of their responses in regions other than in the temperate where most studies on stream processes have been carried out (Ferreira et al., 2020).

Structural and functional indicators may have similar or divergent responses to stressors (Solimini et al., 2006), as evidence shows that changes in ecosystem structure can occur without corresponding changes in function, and vice versa (Ferreira et al., 2020; Gessner & Chauvet, 2002; Sandin & Solimini, 2009; Young & Collier, 2009; Young et al., 2008). Therefore, functional and structural measurements, while focusing on different components, should be used together for a comprehensive ecosystem assessment (Clapcott et al., 2020; Ferreira et al., 2020; Munn et al., 2020; van der Lee et al., 2020; Young et al., 2008).

### Commonly used bioindicators in streams

Most bioassessment tools are based on five Biological Quality Elements (BQEs): macroinvertebrates, fish, macrophytes, and benthic diatoms (EU Commission, 2005; Masouras et al., 2021). These elements are efficient for monitoring anthropogenic impairment in lotic ecosystems, as they respond to different stressors and therefore reveal the ecosystem functioning holistically (Wang et al., 2015). Benthic diatoms are widely used as bioindicators since; they are primary producers, and therefore they form the base of the food web structure (Gulzar et al., 2017; Lobo et al., 2016; Passy, 2007). Diatoms also react to environmental stressors such as nutrient enrichment, organic pollutants, inorganic nutrients, and heavy metals (Marzin et al., 2012). Fish encompass a broad range of trophic levels from primary to secondary consumers enabling them to reflect integrated trophic conditions in an aquatic environment (Anderson & Cabana, 2007). Additionally, their longevity and mobility make them ideal bioindicators of long-term effects (Ormerod, 2003; Souza & Vianna, 2020). Macrophytes are commonly used in heavy metal and pollution-related studies due to their ability to accumulate pollutants and play a significant role in nutrient cycling (Xin et al., 2020), thus are used to assess the distribution and impact of these pollutants (Zhukovskaya et al., 2024).

Most countries have an extensive history of using macroinvertebrates in river ecosystem biomonitoring (Barbour et al., 1999; Birk et al., 2012; Carter et al., 2017). This is because macroinvertebrates are a highly diverse group and are a key constituent of the aquatic food web linking up organic matter and nutrient sources with higher trophic levels (Merritt & Wallace, 2009). Furthermore, they have a sedentary lifestyle thus represents habitat-specific ecological conditions (Carlisle et al., 2014); a relatively longer life span, and subtle life stages enabling them to integrate both long and short-term effects of ecological deviations (Johnson et al., 1993). Additionally, their communities consist of a diverse and widely distributed taxa with a gradient of pollution tolerance and trophic levels providing enough information for interpreting cumulative effects (Bonada et al., 2006). They are also easy to sample (Barbour et al., 1999) and thus, facilitate efficient and effective monitoring efforts.

Additionally, macroinvertebrates provide comprehensive insights into both the structural integrity and functional processes of aquatic ecosystems, making them essential for monitoring environmental health (Harrison et al., 2021; Hladyz et al., 2009). Within the structural or taxonomy-based component, macroinvertebrates have been utilized based on their tolerances to a variety of pollutants and habitat preferences like flow, discharge, water temperature and substrate (Carlisle, Nelson & Eng, 2012;Mellado-Díaz et al., 2019; Min & Kong, 2020; Doychev, 2023). Within the functional or trait-based component, macroinvertebrates have been utilized based on their mobility, feeding preferences, life cycles, indicator trait state, morphological and mobility aspects (Cummins & Klug, 1979; Jiang et al., 2021; Tomanova et al., 2007). Functional traits determine the adaptability and response of macroinvertebrates to gradients of pollution in river ecosystems (Pallottini et al., 2017).

While structural methods have been used for decades, trait-based (functional) methods have proven to be more informative even with coarse taxonomical-level data (Poff et al., 2006). Furthermore, functional methods provide an overview of the underlying mechanisms driving changes in community structure (Liu & Wang, 2018; Verberk et al., 2013). Their response in a predictable manner compliments their use as excellent bioindicators (Mondy et al., 2014).

However, despite these advantages, use of macroinvertebrate functional approaches in the Afrotropical studies is still notably narrow (Masese et al., 2014a, b). This is attributed to shortage of regional basic research, reliance on keys from temperate regions (Tumwesigye et al., 2000; Turyahabwe et al., 2021; Yegon et al., 2021), scarcity of experts (Ochieng et al., 2019) and schemas for classifications and use (Buss et al., 2015). This leads to improper assignment of macroinvertebrates into groups (taxa), behavioral and feeding patterns (Masese et al., 2014a, b). Therefore, there is a need to address these challenges and integrate the functional approaches into monitoring frameworks in the Afro-tropics.

Several African countries are signatories to the UN Sustainable Development Goals, specifically SDG target 6.3.2, which focuses on the proportion of water bodies with good ambient water quality (United Nations, 2015). To fulfill this obligation, countries are required to collect data on five key parameters for global comparison, and countries with laboratories and capacity can provide biological data (UNEP, 2021). The African Union Agenda 2063 promotes environmentally sustainable and climate-resilient economies, with a focus on biodiversity conservation and sustainable natural resources management (African Union,2014). The African Ministers Council on Water has also been formed to strengthen collaboration between ministries of water in Africa. In East Africa, the East African (EA) Vision 2050, PILLAR 3.4 emphasizes the sustainable utilization of natural resources, environmental management, and conservation.

Despite the above provisions, the existing policies and plans do not sufficiently emphasize biomonitoring. For example, Uganda’s National Water Policy 1999 revised 2021, under Water Act 164, and the East African Standard for potable water 2022 focus on physical and chemical parameters, with limited emphasis on biological monitoring. Also, efforts to regulate effluent discharge primarily address physico-chemical, microbiological, organic and inorganic substances aspects, leaving a gap in assessments of ecological health (NEMA 2020). This gap limits the ability of the region to comprehensively predict and manage freshwater ecosystem health, which is essential for sustainable water resource management and biodiversity conservation.

Therefore, research in East Africa, particularly in Kenya (Kitaka et al., 2024; Masese et al., 2023), Ethiopia for the LARIMA project on the Sustainable Highland Rivers Management (Lakew & Moog, 2015), continues to advocate for advanced bioassessment tools, the development of several indices, and capacity development. In Uganda, the Sustainable Water Quality Management Supporting Uganda’s Development Ambitions (SWAQ-Uganda) project aims to develop a biomonitoring framework using bioindicators, especially macroinvertebrates, wadeable streams and rivers.

### Bioassessment and biomonitoring systems in wadeable rivers

#### Global scale

The evolution of biomonitoring has progressed from targeting a single stressor within individual countries to addressing multiple stressors, at the continental level and later collaborating on a global scale. Biomonitoring origins trace back to 350 BC, when Aristotle observed “black mud” and “red tubes” in sewage-polluted brooks, linking these to low oxygen levels and contamination (Moog et al., 2018). This concept evolved further in the 1800 s, as Friedrich Kolenati recognized the absence of caddis larvae in factory-polluted streams (Kitaka et al., 2024; Persoone & De Pauw, 1979). In the 1900 s, Kolkwitz and Marsson (1908, 1909) classified organisms in clean versus polluted waters, introducing the saprobic index to monitor organic pollution with indicators like microalgae, fish, and macrobenthic invertebrates. However, early adoption in countries like the USA and the UK was hindered by views that these methods were specific to Central Europe, their narrow focus on organic pollution, challenges in sampling and identifying diverse organisms, and subjective pollution tolerance categories (Cairns & Pratt, 1993; Persoone & De Pauw, 1979).

With the popularity of the different stressor types, the UK and US developed rapid biomonitoring techniques such as the Trent Biotic Index (Woodiwiss, 1964) and Beck Biotic Index (Beck, 1955) respectively, which assessed organic pollution at the community level and accounted for other environmental stressors. In the UK, tools like the Biological Monitoring Working Party (BMWP, 1978; Chesters, 1980; Paisley et al., 2013) and River Invertebrate Prediction and Classification System (RIVPACS) (Wright et al., 1994; Wright et al., 2000a & b) became crucial in river biomonitoring (Hawkes, 1998). In the USA, the Clean Water Act of 1972 mandated the use of family biotic indices and the incorporation of structural and functional metrics to improve the sensitivity and robustness of biomonitoring programs (Barbour et al., 1999; Hilsenhoff, 1988; Lenat, 1993). Multimetric approaches were introduced to enhance diagnostic capabilities (Cairns & Pratt, 1993; Karr & Chu, 1999).

During the 1960 s and 1970 s, North America began utilizing taxa-neutral diversity indices, providing numerical representations of community composition based on species richness and abundance (Daly et al., 2018). All after which, additional indices emerged, including the Canadian Benthic Assessment of Sediment (Reynoldson et al., 1995), New Zealand’s Macroinvertebrate Community Index (Stark, 1985), the Stream Invertebrate Grade Number Average Level (Chessman, 1995), and the Australian River Assessment Scheme-AUSRIVAS (Davies, 2000). In 2000, the EU Water Framework Directive (WFD) was developed as a legislative structure to implement a uniform standard to monitor and analyze the ecological health of EU surface waters and to maintain or restore them to at least a “good ecological status” (European Commission, 2000).

Water Framework Directive has modified the bioassessments to include Ecoregions, river types, reference site-based, and stressor-specific approaches including hydro morphological degradation (AQEM, 2002; Clarke & Hering, 2006; Hering et al., 2006; Moog et al., 2018). The EU Water Framework Directive (WFD) is extensively applied across multiple regions and countries and serves as an effective model for assessing and standardizing large-scale spatiotemporal diversity, enabling consistent comparisons through intercalibration of aquatic ecosystem assessments (EU, 2024) and despite the significant spatiotemporal variability inherent in freshwater ecosystems (AQEM, 2002; Hering et al., 2006; Birk et al., 2013). However, in 2012, over 300 different bioassessment methods in Europe had been developed, which created challenges for their integration into policy (Birk et al., 2012).

On a global scale, biomonitoring has increasingly become an international collaborative effort. In response, Eriksen et al. (2021) recently proposed a global multimeric, which incorporates three widely used river assessment metrics: BMWP/ASPT, Shannon–Wiener diversity, and EPT richness. In 2022, the IUCN acknowledged that while standardized bioassessments are conducted in regions like Europe (AQEM, 2002; Hering et al., 2006), North America (Barbour et al., 1999; Hilsenhoff, 1988; Lenat, 1993), and Australia (Davies, 2000), similar efforts are lacking in Africa, Asia, and South America yet they are needed to enhance communication and collaboration in addressing globally common environmental and biodiversity loss challenges. Therefore, the IUCN established the Global Task Force on Freshwater Macroinvertebrate Sampling Protocols (GLOSAM)^1^ in the Netherlands to produce standardized guidelines for monitoring freshwater macroinvertebrates, facilitating effective comparisons of ecological issues across regions over time.

#### Africa

In Africa, the first rapid biomonitoring method was developed in South Africa based on the Biological Monitoring Working Party system in the 1990 s (Dallas, 2007; Dickens & Graham, 2002). SASS has undergone several upgrades, the latest being SASS5 (Dickens & Graham, 2002), along with a web-based version, mini-SASS, designed for citizen science (Graham et al., 2004). Mini-SASS has been tested in Kenya; however, it needs rigorous training of the volunteers to be able to give the best taxonomy and classification to achieve good results (Waswala et al., 2019; Kitaka et al., 2024).

SASS has been adapted and tested in most Southern African countries, such as Zambia, Mozambique, and Zimbabwe (Bere & Nyamupingidza, 2014). It has also been modified to develop country-specific benthic macroinvertebrate indices, for example, the Namibian Scoring System (NASS) (Palmer & Taylor, 2004), the Okavango Assessment System (OKAS) (Dallas, 2009), and the Zambia Invertebrate Scoring System (ZISS) (Dallas et al., 2018). Furthermore, the South African Assessment Scheme (SAFRASS) protocol was developed by integrating three bioindicator groups (diatoms, macroinvertebrates, and macrophytes) to enhance the methods’ response to any fluctuations in river settings (Lowe et al., 2013).

In West Africa, Nigeria developed the Niger Delta urban multimetric index (MINDU) in 2019 (Edegbene et al., 2019, 2022), and Togo introduced a Macroinvertebrate-Based Multimetric Index for the Zio River Basin (MMIZB) in 2020 (Tampo et al., 2020). In East Africa, early biomonitoring approaches, particularly in Uganda, were based on BMWP/ASPT, as well as taxa or EPT (Ephemeroptera, Plecoptera, Trichoptera) richness, evenness, and tolerance metrics (Kasangaki et al., 2006, 2008). Later, SASS was tested in Kenya’s Mt. Kenya rivers (M’Erimba et al., 2014) and the Mara River (Oigara & Masese, 2017).

In 2012, the Tanzania Rivers Scoring System (TARISS) (Kaaya et al., 2015) was developed in tropical Africa. TARISS primarily serves Tanzania but has also been tested across East Africa on the Nyabarongo River in Rwanda (Dusabe et al., 2022) and on River Mpanga in Uganda (Tumusiime et al., 2019). Tumusime et al. (2019) in Uganda, found the TARISS taxa list not exhaustive and therefore recommended that TARISS may not apply to all East African rivers and streams unless it is modified and/or validated before its use.

Additionally, some river-specific and area-specific indices have been developed in Kenya, including the Benthic Index of Biotic Integrity (B-IBI) which has been established for monitoring the Moiben River in Kenya (Masese et al., 2023). In Ethiopia, the ETHbios index is used to assess the central and southeast highlands rivers (Lakew & Moog, 2015). Studies in Uganda employ the Rwenzori score for assessing rivers in the Rwenzori regions (Musonge et al., 2020). All these river or area-specific indices are not used on other rivers or areas due to the differences in reference conditions, anthropogenic pressures, and regional species assemblages. However, this can be refined by normalizing the scores from different indices, for example by using the European Ecological Quality Ratio (EQR) can be developed and applied for all indices in all major water body types and ecoregions (EU, 2024). Regional advancement could be accomplished based on long-term monitoring programs and expert decisions for good ecological status thresholds of the EQR. This will allow the development of an assessment system for the entire region.

Research continues to advocate for advanced bioassessment tools in East Africa (Kitaka et al., 2024; Masese et al., 2023). Projects such as the Sustainable Water Quality Management Supporting Uganda’s Development Ambitions (SWAQ-Uganda) aim to develop a biomonitoring framework for assessing lakes and rivers using bioindicators, especially macroinvertebrates.

### Steps towards the development of macroinvertebrate-based river assessment framework in Afrotropical regions

**Fig. 3:**
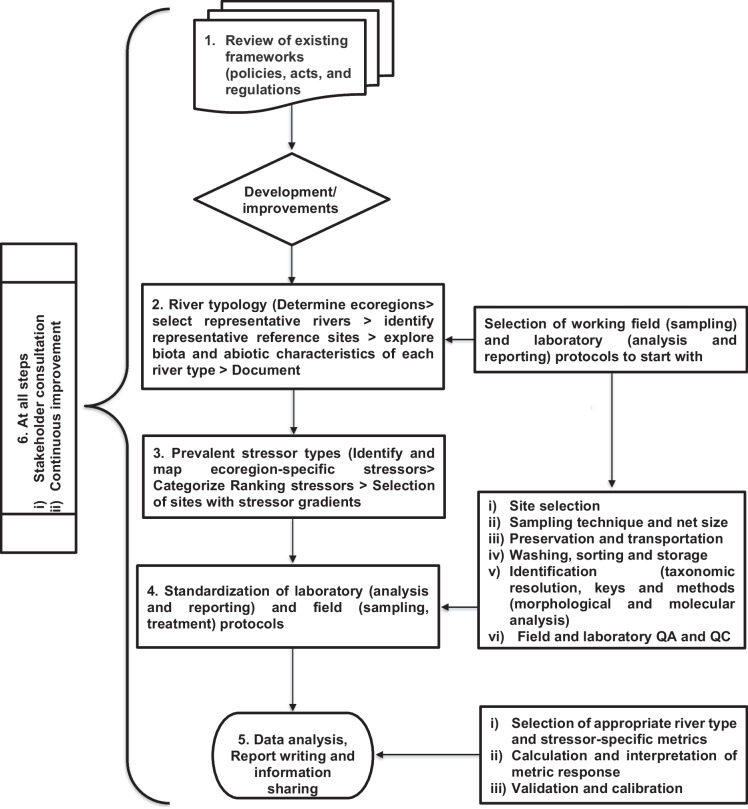
Conceptual diagram on the development of macroinvertebrate-based river assessment framework. The abbreviations are Quality Assurance (QA) and Quality Control (QC)

A national level bioassessment framework intended for policy integration must incorporate an approach that effectively accounts for spatiotemporal variability and adheres to a comprehensive, standardized methodology. With a specific focus on the macroinvertebrate-based bioassessment method, in this section, we propose a systematic, step-by-step procedure, with emphasis on milestones required for the development and implementation of robust river bioassessment frameworks (Fig. 3). It includes the following steps: (1) defining the objective and reviewing the existing frameworks, (2) development of an Ecoregion and river typology, (3) identification of prevalent stressor types and Establishment of Surface Water Monitoring Network, (4) Development of standardized protocols for sampling, laboratory analysis and interpretation, (5) Data analysis and communication—metric selection, calculation, and report writing, and (6) continuous improvement of the framework as detailed in Fig. 3. Subsequently, there must be training of experts and development of quality-checked laboratories to facilitate integration and implementation in national water resources laws.

### Review of existing river assessment frameworks and setting project objectives

In developing a macroinvertebrate-based river assessment framework, the rationale of the project should be consistent with existing national, regional and international ecological management, conservation and water resource development goals. To ensure this, it is essential to carry out a comprehensive review and assessment of existing policies, regulations, frameworks, studies, methodologies, sampling protocols, data analysis and information sharing techniques.

This review process, provides knowledge on existing gaps, limitations, and opportunities for the project’s success. It also forms the foundation for designing and implementing subsequent steps with clearly defined objectives that address spatiotemporal variability and ensure the bioassessment framework is adaptable to different river systems and dynamic environmental conditions.

### River typology development

Developing a river typology at a national level starts with delineating ecoregions followed by documenting the unique eco-hydro morphological characteristics of representative rivers within each ecoregion (AQEM Consortium, 2002; Dodkins et al., 2005; Nijboer et al., 2004). A clear river typology provides a scientific basis for managing rivers within specific ecoregions by considering the ecological, hydrological, and morphological factors. It supports targeted conservation and management strategies tailored to each river type within an ecoregion. The process considers both biotic and abiotic parameters. Key steps include the following:

#### Defining ecoregions

Ecoregions are defined through the grouping of the diagnostic and descriptive features that determine ecological distribution of flora and fauna within a country (Abell et al., 2008; Blasi et al., 2014; Kemp et al., 1999). Data used may include climatic zones, geology, biogeography and vegetation, tectonic regions, landforms and land cover. Ecoregions provide a stratification framework for studying and managing ecological systems (Solheim et al., 2019).

#### Selection of representative rivers

Choose rivers within each ecoregion that reflect its overall characteristics (AQEM, 2002). The selected rivers serve as focal points for the detailed study. They should preferably represent along their continuum all major stressors with a gradient ranging from natural to heavily degraded reaches characteristic of the ecoregion under investigation. However, in defining river types, only reference or near natural reaches are used to represent the expected natural ecological conditions (Nijboer et al., 2004).

#### Defining river types

Two approaches can be used, i.e., the top-down approach, which categorizes rivers based on broader, regional characteristics, and then the second step, a validation using reach-scale biota in the bottom-up approach (Sandin & Verdonschot, 2006). Rivers are then grouped into distinct types based on their unique eco-hydro morphological and biological features, considering factors such as size, flow regime, substrate type, and ecological communities.

#### Documentation of the river typology

Clear documentation of the findings ensures replicability of the study findings, classifications, and methodology used for optimizing river types per ecoregion for future reference, research, and management purposes. A final river typology framework may include but is not limited to per river type characterization of the specific ecoregion, altitudinal range, catchment area range, dominant geological types, expected vegetation types in the catchment, and information on aquatic fauna if available (AQEM, 2002; EPA, 2002; Sandin & Verdonschot, 2006).

### Identification of prevalent stressor types

A principal goal of a biomonitoring system is to detect and quantify degradation to guide effective water resource management (Birk et al., 2012). Once ecological degradation has been detected, decision-makers should determine what specific aspects are to be addressed. Consequently, when formulating a practical assessment method, comprehending the predominant stressors affecting a river system is imperative (Lemm et al., 2021). Furthermore, for a robust assessment framework, stressor-specific evaluation methods and corresponding indices must be established, as the sensitivity and response of biota to different types of stressors have been shown to vary significantly (AQEM, 2002; Sandin & Verdonschot, 2006; Lemm et al., 2021). This should involve the following steps;

#### Categorizing stressors

Stressors may be categorized based on spatial scale, source type or the stressor types. The sources of ecological stressors may be diffuse, i.e., without a single discrete source or maybe point source pollutants with a confined or easily identifiable source (Doychev et al., 2025; Schürings et al., 2024). The common stressors studied include organic matter, nutrient pollutants and toxic substances along with the widespread pressure types such as hydrological modification due to water abstraction, mining, gravel abstraction or flow modification, and morphological impairment due to damming, straightening, sedimentation and the disconnection of the river and its floodplain (Townsend et al., 2008; Hering et al., 2010; Birk et al., 2012). Other uncommon stressors may include pesticides, salinization, pharmaceuticals, micro- pollutants and the often-neglected pressure of physicochemical alteration resulting from temperature regime change derived from river regulation by dams (Doychev & Taneva, 2025; Kampa et al., 2017). In African rivers, land use-related stressors are the most common, including large-scale deforestation or unsustainable riparian land use practice (Alemu et al., 2017).

#### Identifying and mapping stressors within ecoregions or the catchments of selected river systems

The sources of this data could include publications and catchment management plans as well as databases that are already in existence. Furthermore, stressors may also be primarily mapped through surveys and GIS-based materials like satellite imagery (Stoessel et al., 2022).

#### Determining the most important stressors

The categories of stressors may then be associated with spatial scale for further analysis. Different stressors may act individually or superimpose multiple stressors to enhance each other or attenuate their effect (Townsend et al., 2008; Birk et al., 2020). Studies show that river system stressors exhibit uneven distribution, and more so various river types have been found to display distinct responses to different stressors. Following the Driver–Pressures–Stressors–Impact–Response (DPSIR) concept, major stressors at the catchment scale are grouped as pressures and as stressors/state at reach scale.

It is crucial to identify the effects of multiple stressors as their combined action often affects ecosystems differently than individual stressors. These interactions can lead to complex synergistic, antagonistic, additive, or neutral effects on ecological status (Birk et al., 2020). Multiple stressors should be grouped into predefined categories or the four major types, i.e., hydrological (e.g., changes in flow regimes), morphological (e.g., habitat modification), water quality (e.g., pollution), and connectivity (e.g., barriers or disruptions to organism movement) (Schinegger et al., 2011). Additionally, it is important to differentiate the influence of these stressors from natural pressures, such as climatic changes or geomorphological processes. Methods like Social Network Analysis (SNA) can help uncover the structure of co-occurring stressors within datasets, revealing how they interact or co-occur in river systems (Lemm & Feld, 2017).

### Standardization of field and laboratory protocols

Standardization is an essential process to guarantee comparability of the results and involves reaching consensus on procedures for site selection, sampling, sample treatment, sorting, analysis, and reporting (Haase et al., 2004). To ensure effective integration, protocols must define acceptable equipment, habitat choice, sampling intensity, sample processing, and taxonomic resolution, as these factors can influence ecological assessments (Bonada et al., 2006). Protocols can be developed by benchmarking existing guidelines, considering available human resources, equipment access, financial requirements, and the type of river system being sampled. These protocols are then formalized and documented as standards for future reference.

#### Site selection and time of sampling

Standardizing site selection and sampling time is very crucial for consistency and reliable results (AQEM, 2002). These should be primarily informed by the project objectives, alongside the logistical factors mentioned above. Selected sites must provide a good representation of a broad spatial scope along the river stretch and diversity of river types, stressor gradients and habitat diversity (e.g., riffles, pools, substrate types). Also, land use, and the proximity to pollution sources must be considered to capture variability in biological communities. Sampling upstream and downstream of disturbances, like agricultural or industrial discharges, helps to assess human impacts. The time of sampling should account for seasonal variations in biological communities and river conditions. Sampling should coincide with stable environmental periods, preferably during low-flow conditions, to ensure comparability across years and avoid extreme events like floods, which can distort the data.

### Field and Lab methods

**Fig. 4:**
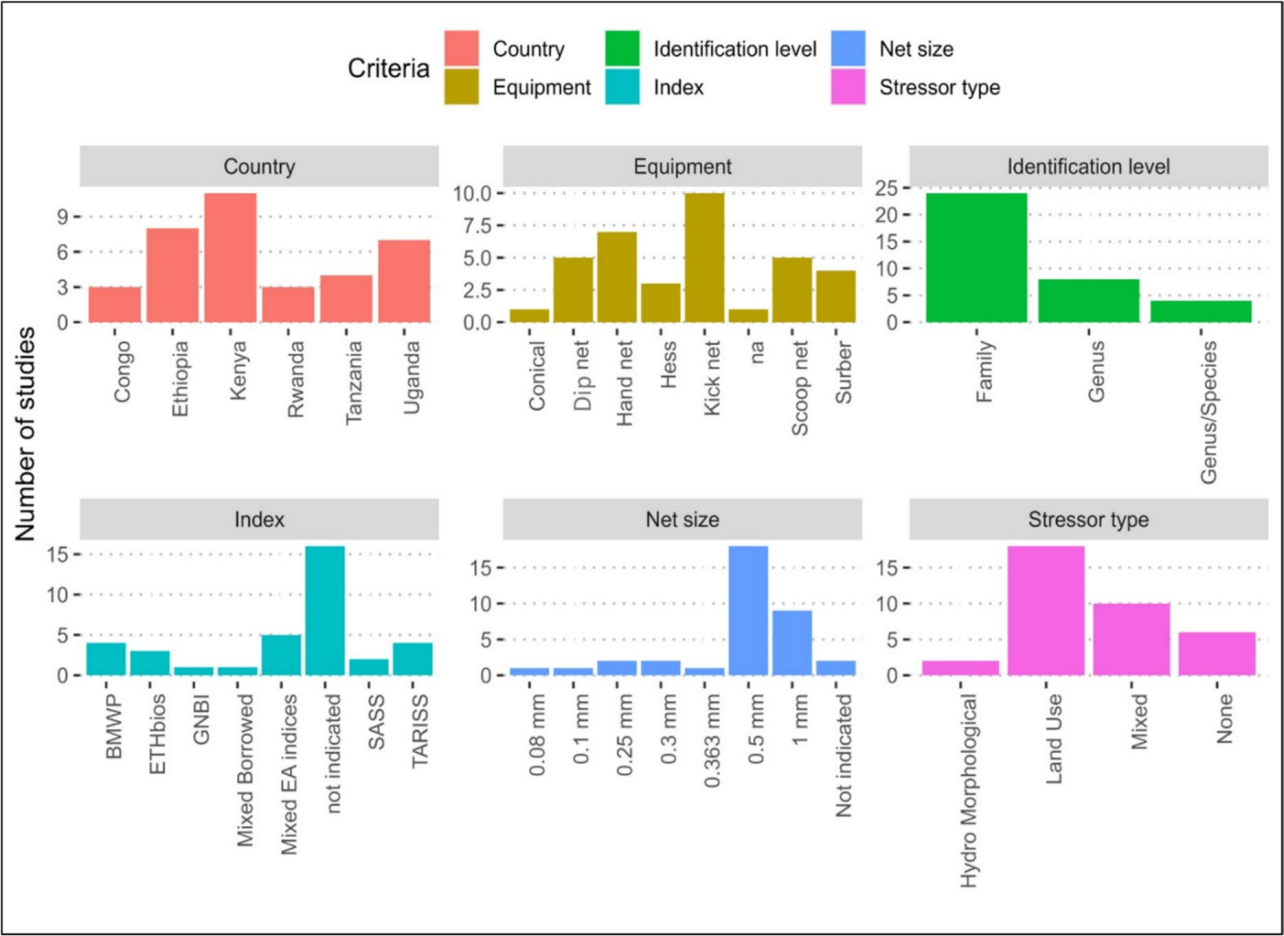
Variability in Biomonitoring approaches across East Africa

Currently in the East African region, there is no standardization of the bioassessment processes. This is evidenced by the different methods used within different countries (Fig. 4 and Appendix 1) To maintain consistency in bioassessment procedures, a preliminary decision should be made on whether to use an area-based sampling method, like the multihabitat approach, which collects samples in proportion to each habitat’s area, or a time-based method, where samples are obtained from each habitat by kicking for a designated time, or potentially a combination of both methods.

Next, then select the appropriate sampler, examples of these include the Conical net, Dip net, Scoop net, Surber sampler, Kick sampler, and Hess sampler for larvae stages and hand nets for adults each with different sampling times and net sizes. The dimensions of the net opening and mesh size used are also critical, finer meshes retain early instar stages, increasing sorting time, while coarser meshes capture larger individuals, resulting in lower densities (Buss & Borges, 2008). Currently, variable mesh sizes are used (Fig. 4), which leads to inconsistencies in results and should therefore, be standardized. East Africa and other tropical nations should adopt standardized mesh sizes for biomonitoring as it is done for other water quality aspects, for example the region’s East African Standards for Potable Water report for 2022. Therefore, establishing a standardized protocol for sorting macroinvertebrates is essential to ensure that all collected samples are accurately and consistently processed across laboratories and projects.

#### Washing and sorting in the laboratory

Laboratory processing of macroinvertebrates involves sieving, subsampling, and identification of organisms. Sieving entails decanting the preservative and washing the sample through stacked sieves of 5000, 3000, 2000, 1000, and 500 µm using high-pressure water splash. Different sieves are often used to separate macroinvertebrates into coarse and fine fractions. The top 4 coarse fractions contain large individuals that may all be counted while the finest fraction contains tiny animals that are usually subsampled. Subsampling involves transferring part of the fine material into a splitter or tray and dividing it into equal squares or units. Hering et al. (2003) recommends that the coarse fraction be subsampled if it contains more than 500 individuals while the finest fraction is subsampled in a 1/16 splitter until 500 individuals are obtained. The abundance obtained after identification and counting is multiplied by the subsampling factor.

During sorting, certain non-target materials such as mollusk shells, empty caddisfly cases, terrestrial taxa, and adult stages of aquatic insects (which may accidentally enter the sample) should be excluded. For damaged organisms, identification should be based on the head or head and thorax, as counting detached legs or abdomens can lead to errors in abundance estimates (AQEM, 2002).

After sorting, a taxa list is compiled using existing literature references relevant to the region. This list includes autecological information on taxa as well as hierarchical classifications at the order, family, subfamily, genus, and species levels, serving as a tool for quality assurance (Hering et al., 2004). However, we acknowledge the absence of a comprehensive identification guide specific to the region. Therefore, identification should rely on guides from the closest regions, supplemented by expertise from the few available taxonomists.

After identification, specimens should be preserved in waterproof plastic vials containing 70% ethanol. The vials should be labeled with the project name, river name, sampling site and date, coordinates, investigator code, sorting date, and taxonomic group. To prevent color loss, samples should be stored in a cool, dark environment (Leitner et al., 2018).

### Identification of Macroinvertebrates

When assessing the ecological condition of freshwater ecosystems, researchers and managers rely on the morphological identification and enumeration of benthic macroinvertebrates to calculate metrics that reflect the overall ecological health and biodiversity of the ecosystem. For a successful robust bioassessment protocol, the target taxonomic level for all taxa must be standardized. However, this is largely dependent on the availability of identification keys, ecological and taxonomic expertise, and supporting infrastructure. Macroinvertebrates exhibit high diversity at lower taxonomic levels, making it essential to conduct localized taxonomic research and develop standardized identification keys.

Key literature on Afrotropical research (Kristensen, 1987; Kristensen & Christensen, 1989; Pennak, 1953; Mandahl-Barth, 1954, 1957; Day et al., 2002; De Moor et al., 2003a, 2003b; Griffiths et al., 2015; Zwick & Zwick, 2023) provides essential resources for identifying common macroinvertebrate taxa down to the family level and, in some cases, to the genus or species level. Although these identification keys are primarily non-tropical, they have facilitated quicker assessments and reduced the level of expertise required in afrotropical river studies. However, identification at the genus and species levels provides more precise data on sensitivity to impairment and ecological relationships. The choice between these levels is often a trade-off between accuracy and practicality, influenced by the availability of financial and expert resources (Schmidt-Kloiber & Nijboer, 2004).

Identification at the species level is crucial when studying specific aspects such as ecology, evolution, physiology, toxicological responses, population dynamics, or secondary production (Bailey et al., 2001; Hjalmarsson et al., 2018). Advancing Afrotropical taxonomy should begin with experts harmonizing and compiling existing resources into systematic keys, which will improve the consistency and accuracy of taxa identification. Developing these keys will ensure that bioassessment methods are both precise and tailored to the region’s unique ecological conditions. However, in East Africa, most studies have identified macroinvertebrates only to the family level, as shown in Fig. 4, underscoring the need for enhanced taxonomic expertise.

The adoption of integrative taxonomy, coupled with the application of molecular macroinvertebrate biomonitoring techniques, offers a promising approach to address the challenges of biomonitoring in East Africa. Molecular methods, such as DNA barcoding and DNA metabarcoding, can complement traditional morphological identification by enabling species-level identification, detecting damaged specimens, and identifying cryptic or immature stages. These techniques are also valuable for identifying taxa from even minimal amounts of DNA, making them a powerful tool in biomonitoring (Hering et al., 2018; Jones, 2008; Leese et al., 2016). Moreover, both bulk sample and environmental DNA studies provide substantial research opportunities, the former because of potential cost efficiencies and the latter because of its capacity to retrieve species DNA from samples of water, sediments, or other material, in the physical absence of the species itself (Leese et al., 2016). Furthermore, metabarcoding has shown a strong positive correlation between sequence read numbers and biomass estimates, making it an effective tool for assessing invertebrate abundance and biomass in ecosystems (Bista et al., 2018; Elbrecht et al., 2017).

In Africa, identification of species using the mitochondrial COI standard marker is successful for certain macroinvertebrate groups, such as Gastropoda (Dusabe et al., 2024; Nalugwa et al., 2010; Plam et al., 2008) and the Plecoptera, especially the Perlidae, subfamily Perlinae (Zwick & Zwick, 2023) because sufficient references in databases exist.

Globally, several databases can be used for species identification, GenBank is the oldest and most comprehensive database maintained by the National Centre for Biotechnology Information (NCBI) and contains all kinds of sequence and genomic information (Benson et al., 2008). However, Genbank is not optimized for taxon identification because it does not require a voucher. Barcode of Life thereafter referred to as BOLD (Ratnasingham & Hebert, 2007), which targets a certain marker as barcode for an organism group; for animals (COI gene or CytB), but also plants (rbcL, matk, 18S), cyanobacteria (16S) and fungi (ITS) (Creer et al., 2016; Hering et al., 2018; Weigand et al., 2019).

BOLD, links one specific barcode sequence to a voucher specimen and thus provides a taxonomic reference. Silva, which is a generalist database that focuses on ribosomal RNA sequences (16S, 18S, 23S, and 28S markers) (Pruesse et al., 2007) and UNITE, which is mainly focused on fungi through the ITS marker (Nilsson et al., 2019).

However, these databases have a strong focus on sequences from North America, UK, Canada, Asia, and Europe, with limited representation from African fauna. This impacts the reliability of African species identification. Additionally, public databases often face issues such as mislabeled sequences, sequencing errors, and low taxonomic resolution (Weigand et al., 2019). This is partly overcome by BOLD with the reference system.

Many countries have developed local fauna reference databases, but these are often unpublished and stored in non-public databases as part of ongoing projects. Some regions, including Europe and North America, have developed country or region-specific DNA reference databases for their respective local fauna, although many of these resources remain unpublished and proprietary (Weigand et al., 2019). In Africa, however, the absence of such resources means scientists must rely on public databases that often lack region-specific data necessary for precise species identification. This reliance can lead to inconsistencies during DNA blast analysis, where a single species may be assigned multiple names at nearly identical similarity levels, complicating identification accuracy.

Therefore, there is an urgent need in Africa, particularly in East Africa, to acquire the necessary resources, including the verified sequences in DNA reference databases with local fauna and related bioinformatics tools or programs. Later, the developed regional DNA database should be linked with public reference databases and aligned to the Global Biodiversity Information Facility (GBIF) to improve on the geographical scale coverage.

In the meantime, the use of Molecular Taxonomic Operational Units (both De Novo and Closed Reference OTUs) or ASVs is possible. ASVs are reproducible across studies and can be applied to future datasets without depending on complete reference databases (Bálint et al., 2024; Callahan et al., 2017).

Environmental DNA (eDNA) has been applied to fish biodiversity monitoring in Tanzania in Rivers and wetlands through the eBioAtlas project^2^; however, this approach is not yet feasible for macroinvertebrates in the region. Furthermore, building capacity in molecular processing and bioinformatics across Africa is key for the success of molecular studies.

### Data analysis—metric selection, calculation, and report writing

#### Selection of appropriate metrics

A metric is defined as an attribute that exhibits empirical changes in value depending on the types of anthropogenic disturbances, such as land use changes or environmental condition changes, within a specific ecosystem (Aura et al., 2017). Common metric types include richness metrics, which measure species variety; diversity metrics that combine species richness and evenness; composition metrics focusing on species dominance; tolerance/intolerance metrics, which assess species’ sensitivity to stressors like habitat degradation and pollution; and abundance metrics that evaluate total population. Each metric reflects a physical, chemical, or biological component of ecosystem quality (Gabriels et al., 2010). These metrics are essential for providing a comprehensive assessment of ecosystem health and are valuable for evaluating the impact of human activities on the environment (Barbour et al., 1999; Macedo et al., 2016).

The selection of appropriate metrics must ensure that they respond to different stressors in a predictable way and are standardized for a robust bioassessment system. This process begins with the identification and development of core metrics, which cover various characteristics of invertebrate communities and fall into four main categories: richness/diversity, composition, tolerance, and functional aspects (Dolédec, 2009). These metrics may consist of both newly developed and established measurements, but to be included in a biomonitoring framework, they must demonstrate a quantitative relationship with stressors. Metric scores can be reported individually or in combination (multimetric reporting) to evaluate the overall health of a stream. A multimetric index, created from a combination of indices, can offer a more holistic view of ecosystem health (Barbour et al., 1996). However, some indices are region-specific, limiting their applicability across multiple regions or countries.

Among the commonly utilized metrics are those that measure species richness, total abundance and the proportion of sensitive taxa groups including Ephemeroptera, Plecoptera, and Trichoptera (EPT) taxa (Emilson et al., 2017; Ofenböck et al., 2010)). Additionally, tolerance metrics like those used in the Biological Monitoring Working Party (BMWP) index (Armitage et al., 1983) and Average Score Per Taxon (ASPT) (Armitage et al., 1983) as well as the percentages of Oligochaeta and Diptera taxa and degradation indices (Ofenböck et al., 2010; Tampo et al., 2021) are commonly used.

Diversity indices, including the Margalef index (Margalef, 1957), Shannon–Weaver diversity and other diversity indices (Shannon & Weaver, 1949; Simpson, 1949; Tampo et al., 2021), similarity indices like Jaccard’s coefficient (Jaccard, 1908) and Sorensen’s coefficient (Ravera, 2001; Sorensen, 1948), functional or trophic measures (Rhithron Feeding Type Index) –RETI (Schweder, 1992), habitat-type preference (e.g., % of clingers), number of (semi) sessile taxa, flow preference measures (e.g., % limnophil, % rheophile, % hypopotamal, and epipotamal zonation index), longitudinal zonation measures, and generation turnover measures (e.g., % bivoltine and % univoltin) (Schmidt-Kloiber & Nijboer, 2004), are among the other diverse array of indices utilized in these assessments.

A biotic index is a quantitative measure used to assess environmental conditions based on the presence, abundance, and diversity of biological communities, particularly in response to pollution or habitat disturbances. Biotic indices are widely favored due to their sensitivity, robustness, cost-effectiveness, and ease of interpretation (Dalu et al., 2016). Rapid Bioassessment Protocols (RBPs) have proven particularly effective for quick evaluations of riverine water quality (Bonada et al., 2006). Examples of biotic indices include the South African Scoring System (SASS), which was the first RBP developed in Africa and has since been adapted and implemented in other Sub-Saharan countries. These adaptations include the macroinvertebrate-based biotic score system (ETHbios) in Ethiopia (Aschalew & Moog, 2015), the Tanzania River Scoring System (TARISS; Kaaya et al., 2015), and the Rwenzori Score (RS; Musonge et al., 2020) (see Fig. 4). Additionally, multimetric indices, such as the Benthic Index of Biological Integrity (B-IBI), have been applied in macroinvertebrate-based assessments in Tanzania (Elias et al., 2014a, 2014b) and Kenyan rivers (Masese et al., 2023), further strengthening bioassessment methodologies in East Africa.

#### Data analysis and interpretation

The ecological status is derived by comparing the biotic community in degraded sites to that in reference sites within an eco/bioregion assessing any deviations that might indicate impairment levels. For macroinvertebrate analysis, calculate each metric’s values and compare them to predefined standards for the specific river/stream types (Barbour et al., 1999). These thresholds should be set conservatively to confirm ecological classes, such as poor, fair, good, very good, or pristine. After computing the metrics and indices, apply appropriate statistical analyses, interpreting the findings according to established objectives and regulatory guidelines. A few softwares have been customized to ease analysis of macroinvertebrate communities during projects; these include AQEM software (Hering et al., 2004; AQEM, 2002), and ECOPROF software (Moog et al., 2018). For the software the taxa list with the count records are uploaded and the different selected metrics calculations made and Ecological Quality Class given. In this case there is need to develop software for the East African region or national states for quicker analysis of metrics.

#### Validation and calibration

The newly developed framework can be validated and calibrated by comparing the macroinvertebrate assessment results with other established measures of river and stream health, such as physicochemical or chemical water quality data or fish community assessments. This will help refine the framework and ensure its accuracy and reliability (AQEM, 2002). Additionally, the stakeholders should be engaged to provide traditional or indigenous knowledge to support the validation of the newly developed framework.

#### Communication and reporting

A clear and concise reporting format to communicate the assessment results to relevant stakeholders such as environmental agencies, researchers, or policymakers should be developed. The results and findings should be presented in an easily understandable manner and provide recommendations for management actions if necessary (AQEM, 2002).

#### Continuous improvement and stakeholder involvement

Establish a feedback loop to incorporate new knowledge, technological advancements, and stakeholder input into the framework. Regularly review and update the framework to improve its effectiveness and adapt it to changing needs and conditions (AQEM, 2002).

It is essential to identify the most relevant stakeholders and understand how they are connected to or impacted by the program. A carefully structured process involving four key steps is recommended as follows: (i) identification of relevant stakeholders. In the context of bioassessment, the stakeholders to consider include policymakers, scientists, environmentalists, and citizens relevant to (1) reporting and enforcing water resource assessment policies across administrative levels; (2) individuals involved in compliance monitoring at lower levels of implementation; and (3) academicians, researchers, and communities directly participating in water quality studies (Nichols et al., 2017). (ii) Analyzing and understanding their perspectives, values, biases, and relevance; (iii) stakeholder mapping, which visualizes relationships between stakeholders; and (iv) prioritizing stakeholders based on their relevance and addressing any potential issues (USAID, 2016).

Feasibly, a diverse group of stakeholders should be engaged at all relevant stages in the development and implementation of a bioassessment system. The process would begin with a summit of policymakers and key scientists to establish objectives, strategies, and priorities for the riverine bioassessment program. This would be followed by the identification of key indicators for assessment, monitoring, restoration, and conservation, alongside the determination of appropriate methods for measuring endpoints. A thorough evaluation of available expertise should then be carried out, along with a needs assessment to guide the development of target infrastructural, financial, skilling, and capacity-building strategies. Finally, a comprehensive implementation and coordination plan should be devised, combining both bottom-up, community-driven bioassessment efforts with top-down, government-led initiatives to strengthen the program’s robustness, adaptability, and long-term sustainability.

Involving stakeholders in the development and implementation of bioassessment programs legitimizes and enhances project acceptance (Verbrugge et al., 2017). It also brings diverse perspectives and expertise, resulting in a more robust and holistic approach that addresses a broad range of ecological, social, and economic concerns (Feio et al., 2021). It helps avoid the misuse of developed methods, redundancy, comparability issues, duplication, and coordination gaps, especially where multiple products already existed in a region (Beck et al., 2020; Kuehne et al., 2020).

## Conclusion

The East African region, though rich in rivers and streams, faces increasing pressures on its water resources due to rapid population growth. This growth is expected to exacerbate issues such as water abstraction, pollution, and infrastructure development, mineral and sand mining. Overdependence on the ecosystem services provided by these water bodies is likely to result in adverse ecological impacts. Currently, the absence of well-documented and systematic bioassessment frameworks for rivers and streams in East Africa hinders the region’s ability to achieve effective ecosystem monitoring. This underscores the urgent need for the development of region-specific bioassessment protocols and indices tailored to the local context. The chronological steps outlined in this review provide a framework for establishing a harmonized bioassessment system, aimed at safeguarding ecological integrity and promoting sustainable management of aquatic ecosystems in the region.

While this review offers a much-needed, systematic pathway for developing bioassessment frameworks in East Africa, certain limitations remain. The proposed approach emphasizes flexibility by advocating for region- and river-type-specific baseline research; however, its success is contingent upon the availability of consistent, high-quality ecological data across countries, something that is currently sparse or fragmented. Moreover, the translation of these guidelines into enforceable policy may face challenges due to varying levels of institutional capacity, funding, and political will across the region. The study effectively outlines a strategic direction, yet the practical steps required to bridge science-policy gaps, harmonize national efforts, and build technical capacity at scale are areas that warrant further exploration. These challenges do not undermine the study’s contribution but highlight key areas for future research and cross-sector collaboration to support its implementation.

## Supplementary Information

Below is the link to the electronic supplementary material.

ESM 1 (DOCX 28.9 KB)

## Data Availability

No datasets were generated or analysed during the current study.

## Footnotes

1 https://www.un-ihe.org/news/new-taskforce-aims-protect-biodiversity-harmonized-freshwater-macroinvertebrate-sampling

2 https://ebioatlas.org/sampling-opportunities/
