# Supplementary material for: Advancements in macroinvertebrate-based river bioassessment research in the Afrotropical region: review and steps towards a regional framework
Source: Environ Monit Assess. 2025 Jul 21;197(8):935. doi: 10.1007/s10661-025-14272-3 (PMC12279587; doi:10.1007/s10661-025-14272-3)
Supplement: Supplementary file 1 — (DOCX 28.9 KB) [file 10661_2025_14272_MOESM1_ESM.docx]

**Supplementary Table 1: Status of biomonitoring in the East African Region**

| **SN** | **Author** | **Title of study** | **Type of sampling equipment** | **Size of sample net** | **Field protocol / macro habitats sampled** | **Time for sampling** | **Season** | **Washing and sorting procedure** | **Stressor type** | **Indices used** | **Guides used** | **Level of identification** | **Country** |
| --- | --- | --- | --- | --- | --- | --- | --- | --- | --- | --- | --- | --- | --- |
| 1 | Tumwesigye et al.,2000 | Structure and composition of benthic macroinvertebrates of a tropical forest stream, River Nyamweru, western Uganda | Hess Sampler | 100 μm | Riffles, pools, debris and vegetation | Not indicated | Wet | No procedure included | None | not indicated | Merritt & Cummins (1978) | Class, order, family, and genus | Uganda |
| 2 | Kasangaki et al., 2006 | Links between anthropogenic perturbations and benthic macroinvertebrate assemblage in Afrontane Forest streams in Uganda | Surber sampler | 363μm | Pools and riffles sampled but microhabitats not mentioned | 1 min | Dry & wet | Procedure not indicated | logging, mining, agriculture | not indicated | Merrit & Cummins (1996), Thirion et al. (1995) | Family level | Uganda |
| 3 | Ochieng et al., 2020 | Comparison of temperate and tropical versions of Biological Monitoring Working Party (BMWP) index for assessing water quality of River Aturukuku in Eastern Uganda | Rectangular kick net of 50 cm *25cm wide aperture | 500 μm | epifaunal substrates (cobbles and gravel), embeddedness, riffles and runs | 2 minutes | Dry & wet | Procedure not indicated | Whole spectrum of environmental Changes | BMWP | Pennak, 1953; Mandahl-Barth, 1954; Merrit and Cummins, 1978; Day and de Moor, 2002; de Moor et al., 2003a, 2003b; Stals and de Moor, 2007. | Class and Family and level | Uganda |
| 4 | Musonge et al., 2018 | Baseline assessment of benthic macroinvertebrate community structure and ecological water quality in Rwenzori rivers (Albertine rift valley, Uganda) using biotic-index tools | Standard hand net | 500 μm | Multi Habitat sampling technique | 5 minutes | Dry | Procedure not indicated | HEP plants, copper-ore exploitation | BMWP | Gerber and Gabriel, 2002 | Family level | Uganda |
| 5 | Fugere et al., 2018 | Ecosystem structure and function of afrotropical streams with contrasting land use. | Surber sampler | 500 μm | Pools and riffles | Not indicated | Dry | Procedure not indicated | Land use changes | Not indicated | Day & de Moor, 2002a, b; Day, de Moor, Stewart, & Louw, 2001; Day, Harrison, & de Moor, 2002; de Moor, Day, & de Moor, 2003a, b; Merritt, Cummins, & Berg, 2008; Stals & de Moor, 2007; Thorp & Covich, 2001 | Family level | Uganda |
| 6 | Tumusiime et al., 2019 | Reliability of the Tanzania river scoring system (TARISS) macroinvertebrate index of water quality: a case study of the river Mpanga system, Uganda | Scoop net of 20 cm diameter | 1000 μm | TARISS protocol; stones, vegetation, and gravel mud and sand | 90 minutes per sampling site | Dry & wet | Procedure not indicated | mining, pollution, agriculture, deforestation | TARISS | Day et al. Citation1999, Citation2002; Day et al. Citation2001a, Citation2001b; Stals and de Moor Citation2007 | Family level and subclass | Uganda |
| 7 | Musonge et al., 2020 | Rwenzori Score (RS): A Benthic Macroinvertebrate Index for Biomonitoring Rivers and Streams in the Rwenzori Region, Uganda | Standard dip net | 500 μm | Multi Habitat Sampling technique | 5 minutes | Dry | Procedure not indicated | HEP plants, copper-ore exploitation | RS, SASS, TARISS | Gerber and Gabriel, 2002 | Family level | Uganda |
| 8 | Bagalwa et al., 2013 | Land use Effects on Cirhanyobowa River Water Quality in D.R. Congo | Hand net | 500 μm | Stones and water plants | 10 minutes | Dry & wet |  | Mining, agriculture, settlement | Not indicated | Needhan and Needham (1962), Micha and Noiset (1982) | Family, Genus, and Species level | Congo |
| 9 | Lohaka et al., 2022 | Preliminary study of the structure of the aquatic macroinvertebrate stands in lake Mai Ndombe in the province of Mai Ndombe in the RDC | conical net | 500 μm | Roots | No time given | Not stated | Procedure not indicated | not stated | Global Normalized Biotic Index (GNBI) | Macan, 1959; Evrard, 2001; Tachet et al.,2006; Mary et al., 2010; Doucet, 2012; Frank et al.,2014 | family level | Congo |
| 10 | Patrick et al., 2015 | Benthic Macroinvertebrates as Indicators of Water Quality: A Case-study of Urban Funa Stream (in Kinshasa, Democratic Republic of Congo) | Surber sampler and hand net | 500 μm | Not stated | Time for sampling not indicated | Dry & wet | Procedure not indicated | Human activities | BMWP, IBGN | Tachet et al., 2006; de Pauw and Vannevel, 1963 | Family level | Congo |
| 11 | Dobson et al., 2002 | Detritivores in Kenyan highland streams: more evidence for the paucity of shredders in the tropics? | modified Hess sampler | 80 μm | Not stated | No time for sampling was stated | Dry | Procedure not indicated | none | Not indicated | Johanson, 1992; Mathooko, 1998; Tachet, Bournard and Richoux, 1980 | Family and genus level | Kenya |
| 12 | Masese et al., 2009 | A preliminary benthic macroinvertebrate index of biotic integrity (B-IBI) for monitoring the Moiben River, Lake Victoria Basin, Kenya | Surber sampler | 250 μm mesh size | Riffles | No time for sampling was stated | Dry | Procedure not indicated | sand harvesting, bathing, laundry washing, grazing | Not indicated | Scholtz and Holm (1985); Johanson, 1992; Merritt & Cummins (1996); Nilson (1996, 1997); Mathooko, 1998 | Genus level | Kenya |
| 13 | Mbaka et al., 2014 | Water and habitat quality assessment in the Honi and Naro Moru rivers,Kenya, using benthic macroinvertebrate assemblages and qualitative habitat scores | Kick net and Hess sampler | 100 μm- for the hess sampler and 1mm for the kick net | Stones, gravel, sand, mud, vegetation, and sediment. | Sediment-3 minutes, Stones in current – 2 minutes, Stones out of current, gravel, sand, and mud -1 minute | Not stated | 0.25mm, 0.5mm and 1mm sieves. | Land use changes (forestry, agriculture, settlements) | SASS | Gerber and Gabriel (2002) | Order and family | Kenya |
| 14 | Aura et al., 2010 | A preliminary macroinvertebrate Index of Biotic Integrity for bioassessment of the Kipkaren and Sosiani Rivers, Nzoia River basin, Kenya | scoop net | 500 μm mesh size | Pools, runs and riffles | No time for sampling was stated | Dry | Procedure not indicated | Agriculture, urbanization | Not indicated | Merritt and Cummins (1997) and Mathooko (1998) | Genus level | Kenya |
| 15 | Masese et al., 2014 | Macroinvertebrate functional feeding groups in Kenyan highland streams: evidence for a diverse shredder guild | Dip net | 300 μm | Riffles and pools | 10 seconds | Dry & wet | Not indicated | Landuse change (forestry, mixed, farmlands) | Not indicated | Gerber and Gabriel (2002), Day and de Moor (2002a), Day and de Moor (2002b), de Moor et al. (2003a), de Moor et al. (2003b) and Merritt and Cummins (2008). | Species, genus, and family level | Kenya |
| 16 | Masese et al., 2014 | Litter processing and shredder distribution as indicators of riparian and catchment influences on ecological health of tropical streams | Dip net | 300 μm | Riffles and pools | 10 seconds | Dry & wet | Not indicated | Landuse change (forestry, mixed, farmlands) | Not indicated | Gerber and Gabriel (2002), Day and de Moor (2002a), Day and de Moor (2002b), de Moor et al. (2003a), de Moor et al. (2003b) and Merritt and Cummins (2008). | Species, genus, and family level | Kenya |
| 17 | Mmerimba et al., 2015 | Monitoring water and habitat quality in six rivers draining the Mt. Kenya and Aberdare Catchments using Macroinvertebrates and Qualitative Habitat Scoring | Kick net and modified Hess sampler | 1mm- kick net; 100 μm- Hess sampler | Vegetation, stones, mud and sand | Time varied depending on the substrate | Not stated | Procedure not indicated | not stated | SASS | Gerber and Gabriel (2002) | Family level | Kenya |
| 18 | Fekadu, 2021 | Impact of land use change in water qualities, habitat qualities and benthic macroinvertebrates assemblages in Kipsinende river, L. Victoria basin, Kenya. | Kick net | 500μm | Riffles, pools, runs and maginal vegetation | 3 minutes | Dry & wet | 300 μm | Landuse change (forestry, mixed, farmlands) | Not indicated | Merritt & Cummins, 2006; Merritt et al.,2008; Merritt et al., 2014 | Genus level | Kenya |
| 19 | Sitati et al., 2021 | Abundance-and biomass-based metrics of functional composition of macroinvertebrates as surrogates of ecosystem attributes in Afrotropical streams | Kick net | 500μm | Gravel, sand mud, stones, and vegetation | 1 minute per biotope | Dry & wet | Not indicated | Landuse change (forestry, mixed, farmlands) | Not indicated | Gerber and Gabriel (2002), Day and de Moor (2002a), Day and de Moor (2002b), de Moor et al. (2003a), de Moor et al. (2003b) and Merritt and Cummins (2008). | Genus | Kenya |
| 20 | Yegon et al., 2021 | Elevation and land use as drivers of macroinvertebrate functional composition in Afromontane headwater streams | Kick and hand net | 500μm | Multi Habitat sampling technique | Not stated | Wet | 0.25mm, 0.5mm and 1mm sieves. | Landuse change (forestry, farmlands) | Not indicated | Gerber and Gabriel (2002), Day and de Moor (2002a), Day and de Moor (2002b), de Moor et al. (2003a), de Moor et al. (2003b) and Merritt and Cummins (2008). | Genus | Kenya |
| 21 | Masese et al., 2023 | Bioassessment of multiple stressors in Afrotropical rivers: Evaluating the performance of a macroinvertebrate-based index of biotic integrity, diversity, and regional biotic indices | Kick net | 1000μm | Gravel, sand, mud, stones inside current. Stones out of current, marginal vegetation | Bedrock and stones inside current: 2-5 minutes, Stones outside current: 1 minute, Gravel, sand, and mud: 1 minute | Dry &wet | Not indicated | Landuse change (forestry, mixed, farmlands) | ETHbios, SASS, TARISS | Gerber and Gabriel (2002), Day and de Moor (2002a), Day and de Moor (2002b), de Moor et al. (2003a), de Moor et al. (2003b) and Merritt and Cummins (2008). | Family level | Kenya |
| 22 | Elias et al., 2014b | Study on freshwater macroinvertebrates of some Tanzanian rivers as a basis for developing biomonitoring index for assessing pollution in tropical African regions | Hess sampler | Not indicated | All available habitats | 30 seconds | not stated | 500 and 100 μm sieves | Agriculture, industries, settlements | Not indicated | Gerber and Gabriel (2002) | Family level | Tanzania |
| 23 | Kaaya et al., 2015 | Tanzania River Scoring System (TARISS): a macroinvertebrate-based biotic index for rapid bioassessment of rivers | 1mm mesh size | 1mm mesh net | SASS protocol (stones, gravel, sand, mud and vegetation) | Time differed based on the biotopes | Dry &wet | Procedure not indicated | human activities & ecosystem stressors | TARISS,SASS | Dickens and Graham 2002 | Family level | Tanzania |
| 24 | Ojija and Lazer, 2016 | Macroinvertebrates as Bio Indicators of Water Quality in Nzovwe Stream, In Mbeya, Tanzania | Dip sampling net | No meh size | Not stated | No time for sampling was stated | Dry | Procedure not indicated | not stated | BMWP | Bouchard, R.W. (2004);Voshell, (2002); Jessup et al.,2002 | Family Level | Tanzania |
| 25 | Mwaijengo et a., 2020 | Seasonal variation in benthic macroinvertebrate assemblages and water quality in an Afrotropical River catchment, northeastern Tanzania | Kick net | 1mm | Stones in current, vegetation, gravel, sand and mud | I minute for stones in current | Dry &wet | 0.5 mm mesh sieve | Landuse change | TARISS | Croft, 1986; Davies and Day, 1998; Gerber and Gabriel, 2002 | Family level | Tanzania |
| 26 | Wronski et al., 2015 | Biological assessment of water quality and biodiversity in Rwandan rivers draining into Lake Kivu | scoop net | 1mm | Pools, riffles, runs, mud, sand and gravel | 90 minutes at each site | Dry &wet | Procedure not indicated | not stated | BMWP | Day et al. 1999, 2001a, b, 2002; Day and de Moor 2002; de Moor and Day 2002; de Moor et al. 2003; Stals and de Moor 2007 | Family level | Rwanda |
| 27 | Dusabe et al., 2019 | Biological water quality assessment in the degraded Mutara rangelands, northeastern Rwanda | scoop net | 1mm | Pools, riffles, runs, mud, sand and gravel | 90 minutes at each site | Dry &wet | Procedure not indicated | agriculture, pastoralism, pollution | TARISS | Day et al. 1999, 2001a, b, 2002; Day and de Moor 2002; de Moor and Day 2002; de Moor et al. 2003; Stals and de Moor 2007 | Family level | Rwanda |
| 28 | Dusabe et al., 2022 | Family-Level Bio-Indication Does not Detect the Impacts of Dams on Macroinvertebrate Communities in a Low-Diversity Tropical River | scoop net | 1mm | Sand, stones, rocks and macrophytes | 60 minutes | Dry &wet | Procedure not indicated | damming | TARISS | Day et al., 1999, 2001a, 2001b, 2002; Day and de Moor, 2002; de Moor and Day, 2002; Stals and de Moor, 2007; de Moor et al., 2003 | Family and order level | Rwanda |
| 29 | Lakew and Moog, 2015 | A multimetric index based on benthic macroinvertebrates for assessing the ecological status of streams and rivers in central and southeast highlands of Ethiopia | Standard hand net | 500μm | Multi habitat sampling technique | Not indicated | not stated | Used sieves of different sizes, not stated | agriculture, urbanization | ETHbios, SASS | not stated | Family and genus | Ethiopia |
| 30 | Aschalew, and Moog, 2015 | Benthic macroinvertebrates based new biotic score “ETHbios” for assessing ecological conditions of highland streams and rivers in Ethiopia | Square frame hand net | 500μm | Multi habitat sampling technique | Not indicated | Dry &wet | Used sieves of 5000, 3000, 2000,1000 and 500 μm mesh sizes | organic pollution, eutrophication, siltation, sand excavation | ETHbios | not stated | Order, family, genus and species | Ethiopia |
| 31 | Gebrehiwot et al., 2017 | Macroinvertebrate community structure and feeding interactions along a pollution gradient in Gilgel Gibe watershed, Ethiopia | Kick net | 250 μm | Different microhabitats | 3 minutes | Dry &wet | Not indicated | damming, urbanization, | Not indicated | RW, 2004, Tachet et al., 2000 | Family level | Ethiopia |
| 32 | Desalegne ,2018 | Macroinvertebrate-based bioassessment of rivers in Addis Ababa, Ethiopia | Surber sampler | 500μm | Pools and riffles | Not indicated | Dry &wet | Sieve with 500 μm | Urbanization, settlements | ETHbios, SASS | Gerber and Gabriel (2002) | Family | Ethiopia |
| 33 | Mezgebu et al., 2019 | Water quality assessment using benthic macroinvertebrates as bioindicators in streams and rivers around Sebeta, Ethiopia | Square frame hand net | 500μm |  | Not indicated | Dry | Sieves of mesh size 2000,1000,750,500 and 250 μm and subsampling procedure indicated | agriculture, industries | Not indicated | Gerber 2002 | Family level | Ethiopia |
| 35 | Lakew ,2020. | Headwater streams and values of bio-assessment for sustainable river management in highlands of Ethiopia | Square frame hand net | 500μm | Multi Habitat Sampling technique | Not indicate | Dry | Sorted using sieves of 5000, 3000, 2000,1000 and 500 μm mesh sizes | agriculture | ETHbios | not stated | Family and genus level | Ethiopia |
| 35 | Lakew ,2022 | Adaptation of field-based ecological health assessment protocol for central and Southeastern highland rivers in Ethiopia | Standard hand net | 500μm | Multi Habitat Sampling technique Pools and riffles | Not indicated | Not stated | Sieves of different sizes used. Sizes not indicated | agriculture, urban, industry | ETHbios | Not stated | Family and genus level | Ethiopia |
| 36 | Getnet et al., 2022 | Macroinvertebrate community structure and diversity in relation to environmental factors in wetlands of the lower Gilgel Abay River catchment, Ethiopia. | D-frame dip net | 500mm | All biotopes present and vegetation | Not indicated | Dry &wet | Not indicated | Land use change (forestry, farmlands) | Not indicated | Gerber and Gabriel (2002); Bouchard (2004) | Family level | Ethiopia |
